# Single-Cell Transcriptomics To Define Plasmodium falciparum Stage Transition in the Mosquito Midgut

**DOI:** 10.1128/spectrum.03671-22

**Published:** 2023-02-27

**Authors:** Mubasher Mohammed, Alexis Dziedziech, Vaishnovi Sekar, Medard Ernest, Thiago Luiz Alves E Silva, Balu Balan, S. Noushin Emami, Inna Biryukova, Marc R. Friedländer, Aaron Jex, Marcelo Jacobs-Lorena, Johan Henriksson, Joel Vega-Rodriguez, Johan Ankarklev

**Affiliations:** a Department of Molecular Biosciences, The Wenner-Gren Institute, Stockholm University, Stockholm, Sweden; b Science for Life Laboratory, Department of Molecular Biosciences, The Wenner-Gren Institute, Stockholm University, Stockholm, Sweden; c Laboratory of Malaria and Vector Research, National Institute of Allergy and Infectious Diseases, National Institutes of Health, Rockville, Maryland, USA; d Population Health and Immunity Division, The Walter and Eliza Hall Institute of Medical Research, Melbourne, Victoria, Australia; e Department of Molecular Microbiology and Immunology, Bloomberg School of Public Health, Johns Hopkins University, Baltimore, Maryland, USA; f Laboratory for Molecular Infection Medicine Sweden (MIMS), Department of Molecular Biology, Umeå University, Umeå, Sweden; g Microbial Single Cell Genomics, Department of Cell and Molecular Biology and Science for Life Laboratory, Uppsala University, Uppsala, Sweden; h Faculty of Veterinary and Agricultural Sciences, The University of Melbourne, Parkville, Victoria, Australia; Hebrew University of Jerusalem

**Keywords:** malaria, *Plasmodium falciparum*, mosquito midgut, scRNA-seq, single cell, stage transition, transmission

## Abstract

Malaria inflicts the highest rate of morbidity and mortality among the vector-borne diseases. The dramatic bottleneck of parasite numbers that occurs in the gut of the obligatory mosquito vector provides a promising target for novel control strategies. Using single-cell transcriptomics, we analyzed Plasmodium falciparum development in the mosquito gut, from unfertilized female gametes through the first 20 h after blood feeding, including the zygote and ookinete stages. This study revealed the temporal gene expression of the ApiAP2 family of transcription factors and of parasite stress genes in response to the harsh environment of the mosquito midgut. Further, employing structural protein prediction analyses, we found several upregulated genes predicted to encode intrinsically disordered proteins (IDPs), a category of proteins known for their importance in regulation of transcription, translation, and protein-protein interactions. IDPs are known for their antigenic properties and may serve as suitable targets for antibody- or peptide-based transmission suppression strategies. In total, this study uncovers the P. falciparum transcriptome from early to late parasite development in the mosquito midgut, inside its natural vector, which provides an important resource for future malaria transmission-blocking initiatives.

**IMPORTANCE** The malaria parasite Plasmodium falciparum causes more than half a million deaths per year. The current treatment regimen targets the symptom-causing blood stage inside the human host. However, recent incentives in the field call for novel interventions to block parasite transmission from humans to the mosquito vector. Therefore, we need to better understand the parasite biology during its development inside the mosquito, including a deeper understanding of the expression of genes controlling parasite progression during these stages. Here, we have generated single-cell transcriptome data, covering P. falciparum’s development, from gamete to ookinete inside the mosquito midgut, uncovering previously untapped parasite biology, including a repertoire of novel biomarkers to be explored in future transmission-blocking efforts. We anticipate that our study provides an important resource, which can be further explored to improve our understanding of the parasite biology as well as aid in guiding future malaria intervention strategies.

## INTRODUCTION

Malaria remains a large global health burden, infecting approximately 224 million people each year and having a death toll exceeding 627,000, mainly children in sub-Saharan Africa (1). Out of the five *Plasmodium* species known to infect humans, Plasmodium falciparum causes the vast majority of severe cases and deaths. *Plasmodium* parasites have a complex life cycle that extends between the human host and the female *Anopheles* mosquito. Parasite development within the mosquito begins during the ingestion of a blood meal containing male and female gametocytes. Gametocytes quickly differentiate into gametes in the mosquito midgut, which fuse to form a diploid zygote. The zygote differentiates into a motile banana-shaped ookinete that migrates within the blood bolus, traverses the midgut epithelium, and forms an oocyst on the basal side of the midgut. During the transition from the blood bolus to the hemocoel, the parasite is exposed to harsh conditions within the midgut, including immune factors from the human host, the midgut microbiome, and oxidative stress from blood digestion (2, 3). Subsequently, the ookinetes lodge into the basal lamina, where they develop into replicative oocysts and thousands of sporozoites are produced. Sporozoites are released from mature oocysts into the hemocoel, from where they invade the salivary glands and are delivered into a vertebrate host when the mosquito takes a blood meal (4).

**FIG 1:**
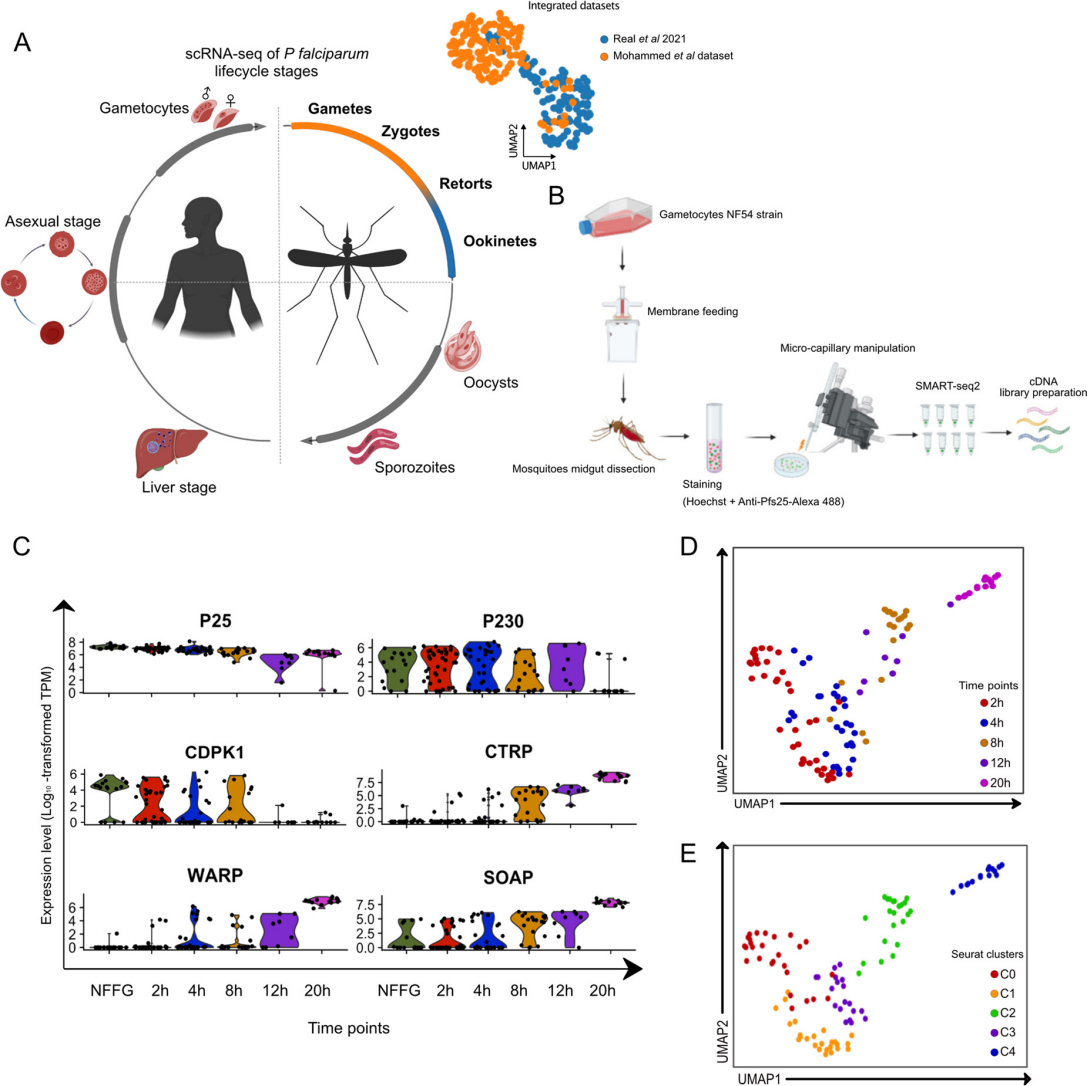
Transcriptomic analysis of single P. falciparum parasites isolated from the midgut blood bolus of A. gambiae mosquitoes. (A) Schematic diagram of the P. falciparum life cycle, highlighting the coverage of single-cell transcriptome data across the life cycle that make up the current malaria atlases. The UMAP shows an overlay of the scRNA-seq data of 24-h ookinetes from the work of Real et al. (16) (blue) and the current study (orange, top right) (illustrations created using www.biorender.com). (B) Schematic diagram of the experimental pipeline: *in vitro*-cultured gametocytes were fed to A. gambiae female mosquitoes by standard membrane feeding. Mosquito midguts were dissected at six different time points, homogenized, and stained, and the homogenate was placed in an inverted fluorescence microscope. Parasites were selected based on their positivity to Hoechst and anti-Pfs25-Alexa 488 staining by fluorescence microscopy and isolated by micromanipulation. cDNA libraries were prepared for each cell using a modified version of the Smart-seq2 protocol. (C) Violin plots of the average expression level (log_10_-transformed transcripts per million) of marker genes for P. falciparum midgut stages. The marker genes were selected from the work of Bennink et al. (18) for validation of our single-cell transcriptome data set. (D and E) UMAP of the single-cell transcriptome data overlaid with the collection time points at which they were isolated (D) or the respective Seurat clusters (E). These plots show the two-dimensional projection of five isolated time points. The global transcriptome similarities and differences were assessed using a k-nearest neighbors (kNN) force-directed graph on the first 10 principal components with true signal variation from our single-cell transcriptome data set based on the Elbow plot.

While P. falciparum undergoes large-scale asexual replication in the human host, sexual recombination occurs only in the mosquito vector. This enables genetic crossing and spread of genetic factors, such as drug tolerance, into the parasite progeny (5). The human-to-mosquito transmission phase is one of the major bottlenecks in the parasite’s life cycle, in part due to the limited number of gametocytes taken up by the mosquito but also due to immune factors from the human blood, the mosquito microbiome, and the effective innate immune response elicited by the mosquito (3). Moreover, the anopheline mosquito has evolved to express a battery of antimicrobial defenses including long noncoding RNAs, nitric oxide, prophenoloxidases, and antimicrobial peptides (6, 7). These events contribute to the large bottleneck in parasite numbers during early development in the mosquito midgut, making these stages a crucial target for transmission-blocking interventions (8). In addition, recent advances in malaria control have bolstered the interest in malaria intervention strategies linked to the reduction of transmission, including vaccines that target sexual, sporogenic, and/or mosquito stage antigens in order to interrupt malaria transmission (9). Although P. falciparum development in the mosquito midgut is considered a prime target for the development of effective transmission-blocking interventions, little is currently known about the transcriptional program that is controlling these processes, with surprisingly few proteins characterized and annotated (10, 11). *Plasmodium* development in the mosquito blood bolus is highly asynchronous, and at later time points a heterogenous population of parasites is found that includes early, mid-, and late ookinetes, as previously shown by Siciliano et al. (12). This asynchrony is a result of the intrinsic biology of the parasite, which results in asynchronous fertilization and consequently asynchronous development, together with the bottleneck imposed by factors from the human and the mosquitoes that affect parasite development (reviewed in the work of Smith et al., 2014 [3]). Therefore, bulk RNA sequencing is not an appropriate approach to study the development trajectory of the parasite, let alone transcriptional heterogeneity between parasites at a given time point. In recent years, single-cell RNA sequencing (scRNA-seq) has been used extensively to resolve cellular heterogeneity, discern key transcriptionally regulated biological processes, and describe transcript and protein expression patterns and functions in multiple stages during the life cycle of P. falciparum (13–17). Nonetheless, a detailed and comprehensive transcriptional map of key P. falciparum developmental stages in the mosquito midgut is still missing (Fig. 1A).

In this study, we use scRNA-seq to explore the P. falciparum developmental dynamics from unfertilized female gametes through the zygote and the ookinete stages. All parasite cells were carefully isolated by micromanipulation from infected Anopheles gambiae mosquitoes. We analyzed the timing of expression of genes connected to biological processes critical for parasite development in the mosquito midgut, including cytoskeletal modifications, invasion, and meiosis. Computational analyses identified the timing of expression for members of the ApiAP2 family of transcription factors throughout the developmental timeline. Further, expression of a set of genes containing the upstream binding motif of the stage-specific ApiAP2-O was connected to the expression of this transcription factor. Moreover, we provide insight into the connections of the parasite’s stress responses to the host environment during development in the mosquito midgut. Finally, we identify several highly expressed genes with nonannotated function that are predicted to be intrinsically disordered proteins (IDPs). The elucidation of genetic factors involved in P. falciparum’s differentiation in the mosquito midgut provides vital insights into the biology of these vulnerable developmental stages while identifying new targets for the control of malaria transmission.

## RESULTS

### Unsupervised clustering defines a developmental trajectory and transcriptional heterogeneity among the early developmental time points.

To enable high-resolution transcriptional profiling of P. falciparum as it develops in the lumen of the mosquito midgut, we infected the African main malaria vector A. gambiae with mature stage V gametocytes of the NF54 genetic background. Mosquito midguts were dissected at 2, 4, 8, 12, and 20 h postinfection (p.i.) and homogenized to release the blood bolus content, followed by Hoechst (DNA) and anti-Pf25 antibody staining to label the parasite sexual stages (female gametes, zygotes, and ookinetes). Parasites do not develop synchronously in the midgut of the mosquito (12), and therefore, we sought to visualize parasites prior to isolation through DNA and Pfs25 staining and to select only parasites with intact morphology. Individual parasites were collected by microcapillary manipulation within 30 min after dissection and homogenization (Fig. 1B), and cDNA libraries were prepared for each isolated parasite using a modified version of the Smart-seq2 protocol (14, 17) using 24 cycles for cDNA amplification.

A total of 180 single cells were collected, of which 125 single cells were retained for subsequent analyses after filtering out cells with poor read counts. The remaining cells were quality controlled based on mRNA count and number of genes expressed (see Fig. S1A in the supplemental material). The data from each time point were validated by analyzing the expression levels of genes previously shown to have a role in parasite development in the midgut. We included genes which have been shown to be expressed early in parasite development, including *p230*, *CDPK1*, and translationally repressed *P25* (18). The expression of these genes in our data set was confirmed to be highest in the early stages with a gradual reduction as development continued (Fig. 1C). Genes associated with ookinete development or midgut invasion, like *CTRP*, *WARP*, and *SOAP*, showed significantly elevated expression levels toward the end of the time course (Fig. 1C). Since our data set represents a subset of cells that have not been previously sequenced, we integrated and normalized our data with a recent single-cell data set that largely covers all other stages of parasite development within the mosquito (16). Comparing our data set with that of Real and colleagues (16), we could validate our coverage of early *Plasmodium* developmental stages. As seen in Fig. 1A, our late, 20-h time point aligns with the majority of cells from the Real et al. data set, which were collected at 24 h after mosquito infection, while our earlier time points form separate clusters (Fig. 1A and Fig. S1B).

A total of 2,000 genes with significant variance were selected for downstream analyses, while 2,835 did not show significant variance (Fig. S1C). Further, among the genes with the highest average change, we found several nonannotated genes and genes that were previously shown to have essential roles during parasite development in the midgut, such as *GAMER*, *PIMMS1*, *PIMMS57*, and *SOPT* (Fig. S1D) (19, 20). Next, we clustered the cells in an unsupervised manner using uniform manifold approximation and projection (UMAP) nonlinear dimensionality reduction, which identified five distinct clusters that largely correspond to the time points at which parasites were collected (Fig. 1D and E; see Materials and Methods for details). The UMAP displays transcriptional variability between and within isolated time points. Early time points, in particular, were computationally predicted to diverge by cell type within each time point (Fig. S2A).

### Pseudotime alignment indicates heterogeneity among the early time points.

**FIG 2:**
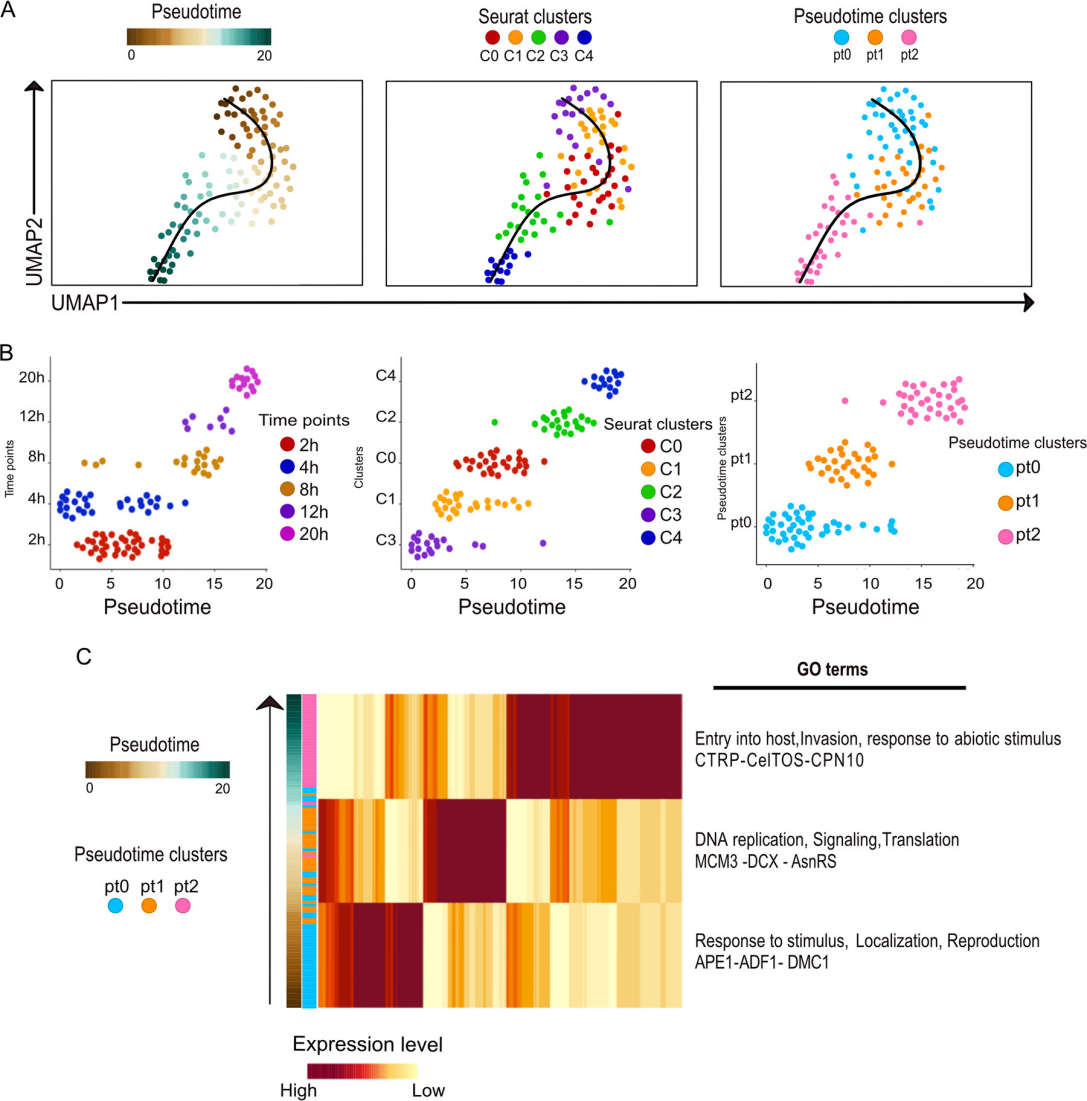
Ordering cells based on the developmental trajectory. (A) Ordering of cells along their pseudotime developmental trajectory using Slingshot. (Left) Pseudotime alignment of cells used in this study, where each dot represents an individual parasite and their positioning is determined by the total relative expression in comparison to the other parasites included in the analysis. The color coding represents their predicted development along the pseudotime axis. (Middle) The pseudotime developmental trajectory overlaid with the assigned Seurat clusters, indicating that C3 is at the root of the trajectory and C4 represents the terminal state. (Right) The pseudotime line overlaid with three unique mRNA patterns based on overarching similarity among major cell communities formed during the process of lineage reconstruction. (B) Slingshot ordering of cells plotted over a pseudotime axis. (Left) Cells from each collection time point plotted on the pseudotime axis. (Middle) Cells representing each of the five Seurat clusters plotted on the pseudotime axis. (Right) Pseudotime clusters plotted on the pseudotime axis. (C) Heatmap showing the average transcript abundance across the pseudotime (pt) axis, based on the Slingshot clustering (pt0, pt1, and pt2). Notable GO terms are indicated for each of the three pt clusters.

Given the heterogeneity of the identified cells, we sought to determine a biologically relevant temporality to the cell clusters. Since the nonfertilized female gametes (NFFGs) were cultivated *in vitro* and therefore not exposed to stresses from the mosquito midgut, we performed a cell trajectory pseudotime analysis that included NFFGs. The results indicated that the inclusion of these cells created a second trajectory, which appeared to distort the natural developmental trajectory as visualized in Fig. S2B. Therefore, we excluded the 0-h (*in vitro*) time point from subsequent pseudotime analyses. To infer a pseudotime alignment for the five clusters identified by Seurat analyses, we reconstructed the cell trajectory in terms of their relative developmental stage. Clusters were reordered with C3 indicated to be the earliest developmental stage, followed by C1, C0, and C2 and ending with C4 as the terminal cluster (Fig. 2A). Further, when the data were ordered along a temporal plane, i.e., the pseudotime (pt) axis, three distinct clusters, pt0, pt1, and pt2 (Fig. 2A, right), were delineated. The pt lineage showed general similarity with the sampling time points, although the early time points showed a trend of asynchronicity where some of the cells aligned with a more progressed stage of development while others aligned with a differing transcriptional state, which we hypothesize may indicate stalled development (Fig. 2B, left). Further, the Seurat clusters showed greater spread over the pseudotime within a single group and more overlap between groups in comparison to the pt clusters, which were more distinct (Fig. 2B, middle versus right). While the pt clusters showed more mutual exclusivity than Seurat clustering, the Seurat clustering indicated more distinct intermediary points in the transition from the early zygote to the ookinete. We utilized the Seurat cluster variance when exploring the role of key genes in transcriptional programing while we relied on Slingshot pseudotime clusters for determining distinct biological processes. The Gene Ontology (GO) enrichment for the pt clusters revealed GO terms similar to those of the Seurat clustering, pointing to an ordered and predictable transcriptional regulation of gene expression programs. For example, pt0 was related to “response to stimulus,” “localization,” and “reproduction” and pt1 was related to “DNA replication,” “signaling,” and “translation,” while pt2 was related to “entry into host,” “invasion,” and “response to abiotic stimulus” (Fig. 2C). To identify the genetic signature for each cluster, we generated the scaled average expression of the top 10 differentially expressed genes for the Seurat clusters (C0 to C4) and the top 20 differentially expressed genes for the pt clusters (pt0 to pt2) (Fig. S3A and B, respectively). Thus, based on GO term analyses, clusters C3 and C1 align with pt0 and represent early zygote development and C0 aligns with pt1 and represents the maturing zygote to early ookinete development, whereas C2 and C4 align with pt2 and represent mid- to late ookinete development.

### Pseudotime ordering identifies gene programs that drive P. falciparum development in the midgut.

**FIG 3:**
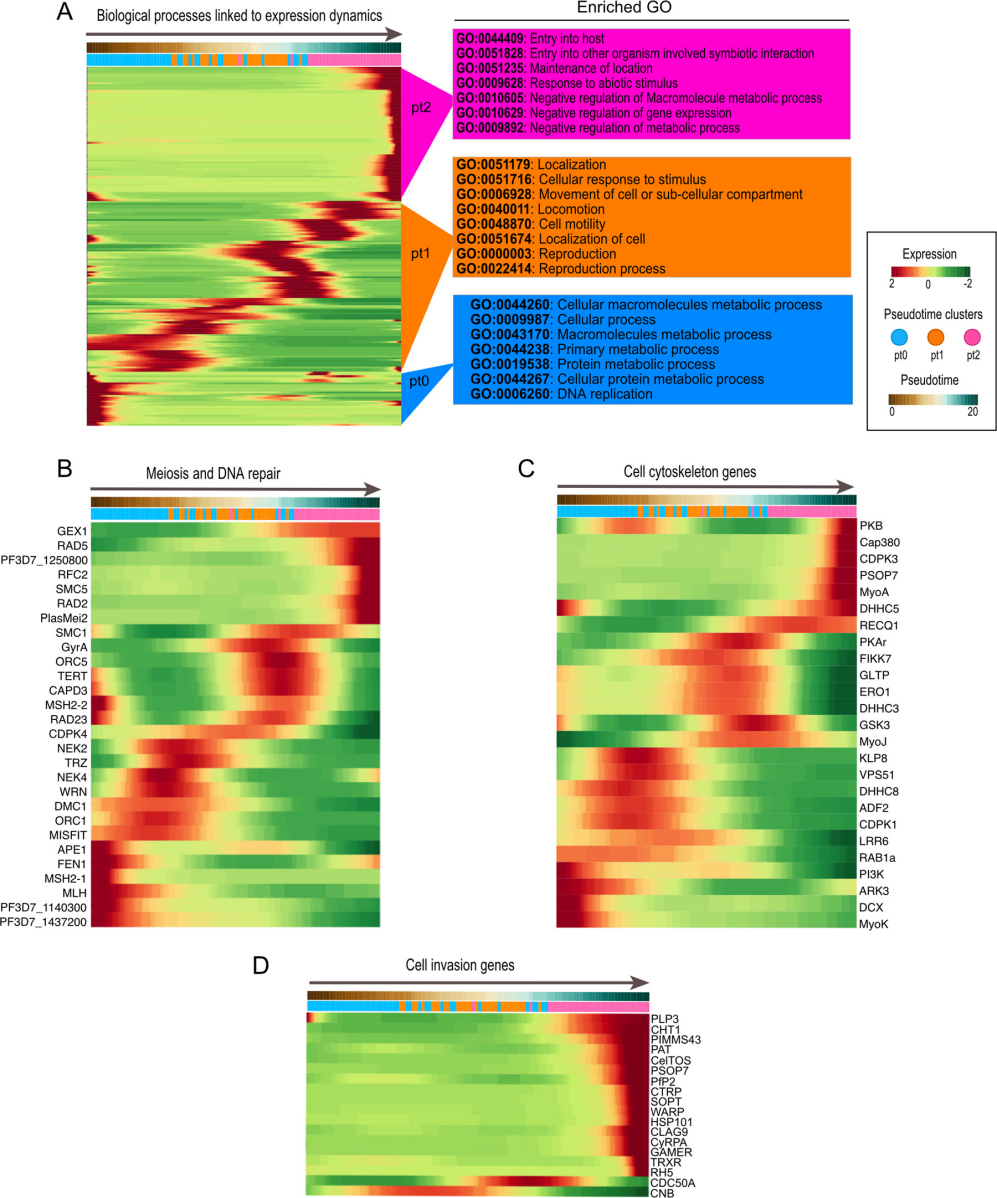
Top differentially expressed genes across the pseudotime axis. (A) Hierarchical clustering of the top 200 expressed genes across the Slingshot pseudotime axis. The genes were grouped according to their ontology terms and analyzed for the top GO terms corresponding to each pseudotime cluster, indicated to the right of the heatmap. (B to D) Heatmap of genes directly or indirectly involved in meiosis and DNA replication (B), cytoskeleton remodeling (C), and cell invasion (D) across the Slingshot pseudotime axis. For all heatmaps, genes were selected based on their biological profile and annotation in PlasmoDB and clustering was based on the Euclidean distance of scaled rows.

To gain insight into the transcriptional programming for the clusters identified over the pseudotime, we performed hierarchical clustering and gene ontology analysis of the top 200 differentially expressed genes across all time points (Fig. 3A; Table S1). We observed a sequence of biological events corresponding to the development from early zygote to ookinetes over the pseudotime, as supported by the top-scoring GO terms which included “DNA Replication” in pt0, “Reproduction” in pt1, and “Entry into host” and “Negative regulation of metabolic process” in pt2. After we performed a gene set enrichment analysis over the pseudotime, increasingly fewer cells showed enrichment for metabolic processes. The average expression of each significantly upregulated program was assessed throughout the developmental time course (Fig. S4A and Table S2). To further define patterns of biological processes among the gene modules that make up the pseudotime clusters, we also ran overrepresentation analyses of the pt clusters’ (pt0 to pt2) gene ontology terms (Fig. S4B, C, and D and Table S4).

In addition, we wanted to further define the timing of expression of specific gene families known to be essential during P. falciparum development in the mosquito midgut (see Table S6, which links gene names with the corresponding gene identifiers [IDs] of genes described throughout this article). Given that fertilization and meiosis occur in the midgut, we investigated the stage-specific expression of known P. falciparum meiosis and DNA repair genes and used the pseudotime analyses as a baseline to gain insight into the timing of expression of these processes during parasite development in the midgut (Fig. 3B). A distinct set of early-expressed genes, including *APE1*, *FEN1*, *MSH2-1*, *MLH*, *PF3D7_1140300*, and *PF3D7_1437200*, were included in DNA repair and DNA replication. A subsequent set of genes that correspond to developmental progression through meiosis in the early zygote included the expression of *MISFIT* (male-inherited sporulation factor important for transmission), *FEN1* (a DNA repair enzyme), *DMC1* (a meiotic recombination protein), and *MLH* (a DNA mismatch repair protein). *MISFIT* may also contribute to cell cycle progression and replication dynamics in conjunction with *NEK4* and *ORC1*, leading to the development of the early ookinete (21). A third set of genes expressed at an even more advanced stage of development included *RAD23* (involved in DNA repair) and *GyrA* (DNA topoisomerase activity), as well as *MSH2-2* (involved in DNA recombination), *Capd3* (meiotic chromosome condensation), *TERT* (telomere maintenance), and *SMC1* (involved in sister chromatid segregation). In summary, the high-resolution single-cell data set enables the description of expression timing of key genes involved in meiosis and its coordination. Interestingly, we observe a difference between *ORC1* and *ORC5* expression, which may indicate how the timings of the different subunits of the origin recognition complex in P. falciparum are orchestrated in their expression.

Towards the end of the pseudotime, there are seven genes, including *RAD2*, *RAD5*, and *PF3D7_1250800*, involved in DNA repair that may be expressed in response to oxidative stress caused by heme released from hemoglobin digestion or alternatively expressed in late ookinetes in preparation for the subsequent mass replication during sporogony which takes place in the oocyst. The additional four genes *RFC2* (involved in DNA replication), *SMC5* (structural maintenance of chromosomes), *GEX1* (karyogamy), and *PlasMei2* (linked to schizogony) are likely to function during sporogony in oocysts.

Considering the extensive morphological changes that occur during P. falciparum development from early zygotes to mature ookinetes, we sought to better understand the coordination of its cytoskeletal gene network (Fig. 3C). We found that *ADF2*, *MyoK*, and *DCX*, involved in actin depolymerization, cytoskeletal regulation, and protein polymerization, respectively, were highly expressed in pt0, as were *Pi3k* and *Rab1a*, indicating a potential role for endosome recycling in the early development of zygotes. Pt1 showed upregulation of *VPS51* and *DHHC8*, potentially increasing vesicle sorting and protein transport, followed by the upregulation of *DHHC3*, which is involved in protein palmitoylation, and *FIKK7.1*, involved in protein phosphorylation and signal transduction. Thus, pt1 likely portrays the coordination of vesicles and protein transport that aids in parasite survival in the gut and development from early to mid-/late ookinetes. Finally, cells in pt2 significantly expressed additional regulators of actin (*ADF2*) and cell division (*MyoJ*) as well as ATP-dependent DNA helicase Q1 (*RECQ1*), which plays a role in DNA double-strand break repair and DNA unwinding (22), possibly in preparation for the subsequent sporogony. Further, genes encoding late ookinete proteins like *Cap380* (oocyst capsule protein), essential for oocyst formation (23), and *PSOP7* (putative secreted ookinete protein), linked to midgut epithelial invasion (24), were both highly expressed in preparation for epithelium invasion and subsequent oocyst development (Fig. 3C).

Additionally, we analyzed the timing of expression of ookinete invasion genes, which are almost exclusively expressed within pt2. These include well-known ookinete marker genes such as *Gamer*, *SOPT*, *WARP*, and *PIMMS43* (Fig. 3D). Interestingly, *Clag9*, a cytoadherent molecule, during erythrocyte invasion and nutrient uptake (25), was found to be upregulated in late ookinetes.

### Defining the temporality of expression of ApiAP2 family members.

**FIG 4:**
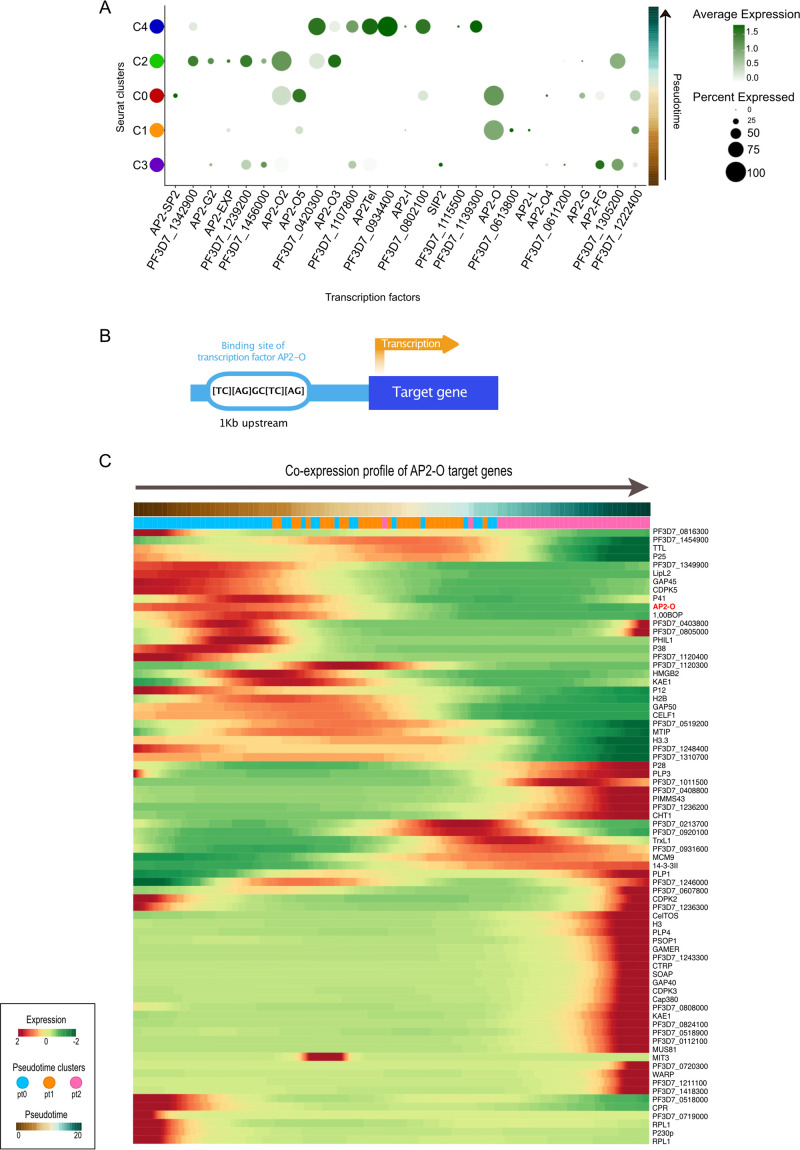
Gene expression patterns of the ApiAP2 transcription factors throughout the P. falciparum development in the mosquito midgut. (A) Dot plot showing the average expression and the proportion of cells that express each of the 27 ApiAP2 transcription factors across the Seurat pseudotime ordered clusters. (B) Schematic illustration of the process of validating genes harboring an ApiAP2-O binding site, including a motif screen of the 1-kb upstream region from the transcription start site of select genes. (C) Pseudotime heatmap demonstrating the timing of expression of the ApiAP2-O transcription factor and its putative *cis*-target genes. Scaled expression values of the genes were ordered based on high (red) or low (green) differential gene expression across the pseudotime axis.

Members of the ApiAP2 transcription factor family, which in P. falciparum consists of 27 genes, regulate differentiation and stage progression events (10). We observed significant upregulation of expression in four out of the five *AP2-O* genes expressed during parasite development in the mosquito midgut; *AP2-O4* did not show expression at any time point (Fig. 4A). *AP2-O* was found to be expressed in the C0 and C1 clusters, indicating high expression in the early zygote and in agreement with a previous report showing *AP2-O* transcription in stage V female gametocytes (26). *AP2-O2*, *AP2-O3*, and *AP2-O5* show expression in C0 and C2 (Fig. 4A), during the zygote-to-ookinete transition. Thus, the timing of expression of the *AP2-O* genes supports a role in the regulation of genes linked to ookinete function, as previously shown by functional analyses (27–29). Interestingly, we observe additional *AP2* genes that appear to be upregulated in a significant proportion (>50%) of cells within their respective clusters (Fig. 4A). These include two additional *AP2* genes in C2 (*PF3D7_1239200* and *PF3D7_1342900*) and four AP2 genes in C4 (*PF3D7_0420300*, *PF3D7_0934400*, *PF3D7_0802100*, and *PF3D7_1139300*), none of which has previously been linked to any developmental stage. In agreement with our data, *PF3D7_0934400* was shown to be highly expressed among ookinetes in the single-cell data by Real and colleagues (16). Also, *PF3D7*_*0622900*:*AP2Tel*, which is significantly upregulated in C4, appears to show low to intermediate expression among ookinetes in the same data set (16).

To gain further insight into gene regulation during P. falciparum zygote-to-ookinete development, we made use of previously published chromatin immunoprecipitation sequencing (ChIP-seq) data on Plasmodium berghei parasites to identify genes regulated by *AP2-O* (26). The target genes from this study were used to find orthologs in the P. falciparum genome that showed significant upregulation in our scRNA-seq data. To validate the orthologs, we investigated the presence of the AP2-O binding motif [TC][AG]GC[TC][AG] (10, 26) within a 1-kb region upstream of the start codon of differentially expressed genes (Fig. 4B). We found that a small number of genes putatively regulated by *AP2-O*, including *p12*, *PF3D7_1349900*, *PF3D7_0518000*, and *cpr*, were upregulated at the beginning of the pseudotime followed by downregulation at a time point earlier than or similar to the expression of *AP2-O* (Fig. 4C). The *p12* gene belongs to the 6-cys protein family expressed in blood-stage parasites and was detected in the proteome of merozoites (30). The *cpr* gene has been predicted to encode NADPH-cytochrome P450 reductase, with a predicted role in oxidation-reduction (GO:0055114, AmiGO, geneontology.org). PF3D7_1349900 and PF3D7_0518000 are both unknown proteins but have previously been shown to be transcribed in mature gametocytes and to some level in ookinetes (31).

To compare the genes expressed in our scRNA-seq data set and containing the AP2-O binding motif with the orthologs in the study by Kaneko and colleagues (26), we included only genes expressed by at least 25% of the cells in each pt cluster. Due to the nature of dropouts in single-cell sequencing data and the conservative threshold set in our analysis, our list of genes is not as extensive as the one made by Kaneko et al. (26). However, our analysis shows a significant overlap with the target genes validated in the work of Kaneko et al. We detect significant expression of genes involved in midgut epithelium invasion and oocyst formation, including the secreted proteins CelTOS, CHT1, CTRP, GAMER, PSOP1, SOAP, and WARP, the plasma membrane-associated protein P25, the oocyst development protein Cap380, and microneme proteins PLP3 and PLP4. Furthermore, the pellicular protein TTL, histone proteins H2B, H3, and H3.3, and DNA replication proteins MCM9 and CDPK3, which are essential for midgut infection, are all expressed in our data set. Finally, PIMMS43, previously described as having a function in parasite immune evasion and sporogonic development (20), and MUS81, involved in DNA repair (32), were also expressed.

### Response to stress during mosquito midgut development.

**FIG 5:**
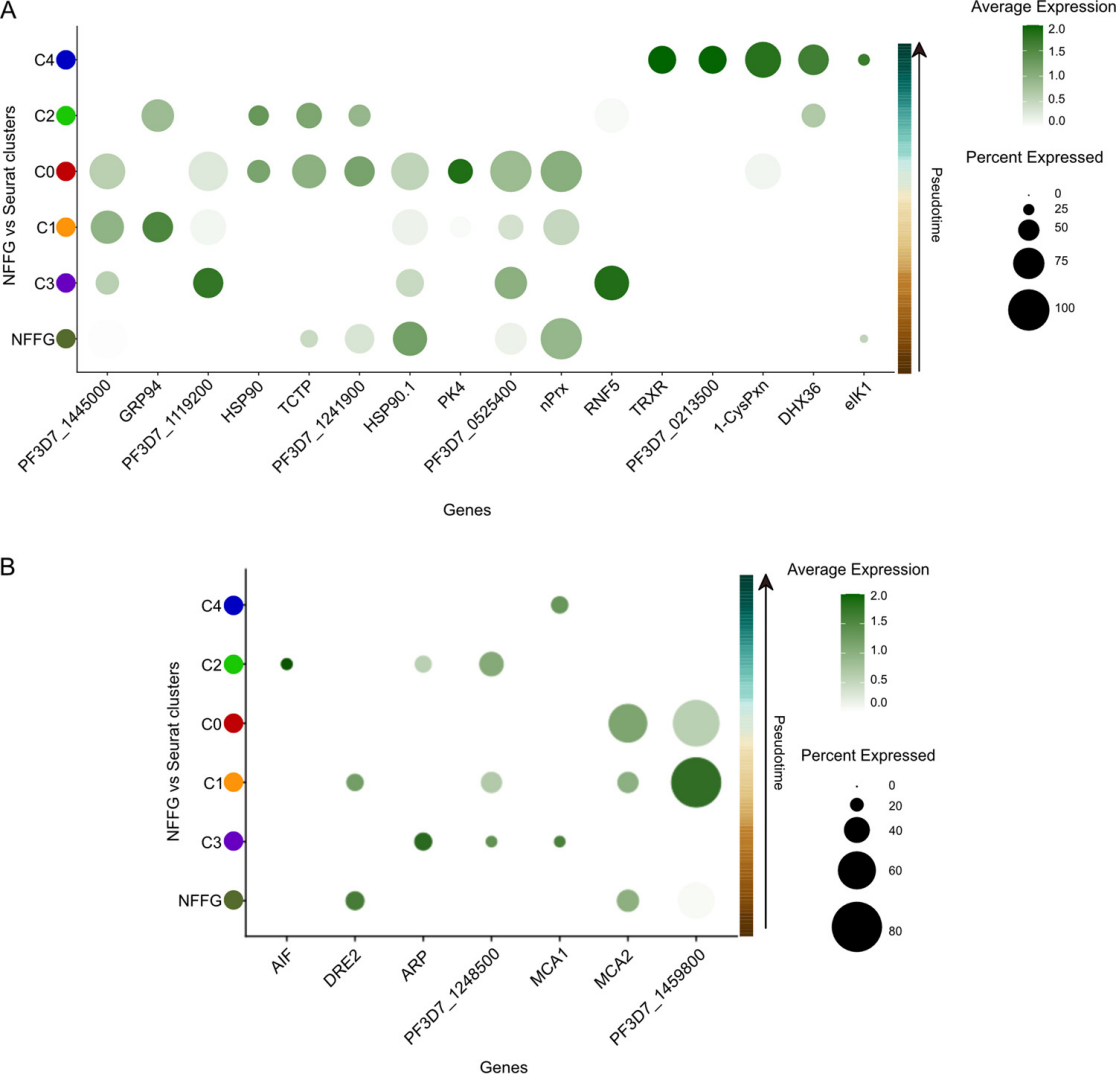
Timing of expression of genes linked to parasite cellular stress and apoptosis. (A) Dot plot comparing the average expressions of stress response genes among pseudotime ordered cell clusters (postinfection) versus nonfertilized female gametes (NFFGs) isolated directly from cell cultures. (B) Dot plot comparing the average expressions of apoptosis-related genes between pseudotime ordered postinfection cell communities inside the mosquito and NFFGs.

The stress response elicited by P. falciparum is an essential mechanism for adaptation to host environmental factors across the life cycle (33). We investigated the transcriptional activity of known cellular stress marker genes and found transcriptional differences between the Seurat clusters, C0 to C4 (Fig. 5A). The cells in the C0 cluster showed higher average expression of *nPrx*, which encodes a nuclear peroxiredoxin that has been suggested to protect nuclear components from oxidative damage or be involved in DNA repair during the asexual blood stage (34). Further, genes such as *TRXR*, *PF3D7_0213500*, 1*-CysPxn*, and *DHX36* are specifically expressed during the later developmental stages, C4 (Fig. 5A). We also compared the stress genes in NFFGs, which showed a smaller population of cells expressing stress-related genes than did C0 (Fig. 5A and B). The genes expressed in *in vitro*-generated NFFGs likely include genes expressed through a preprogrammed default stress pathway in comparison to the larger number of heterogeneous genes expressed in C0. The population of cells in C0 likely demonstrates two trajectories for either cells fated to properly mature into an ookinete or parasites that arrested and failed to mature.

### Protein modeling of stage-specific nonannotated genes.

**FIG 6:**
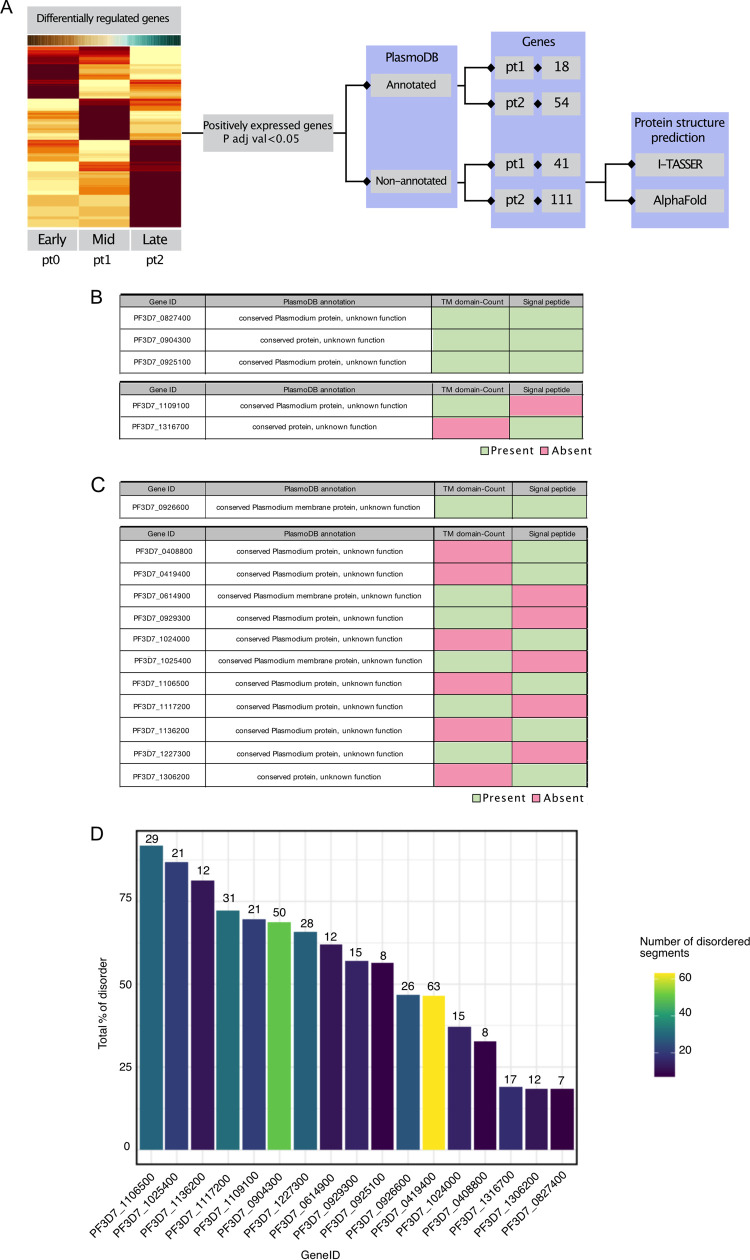
Structural predictions of putative therapeutic targets. (A) Flow diagram depicting the selection process of highly expressed, nonannotated genes. Genes with adjusted *P* values of <0.05 and Bonferroni adjustment (FDR of <0.05) were selected in pt1 and pt2 and further analyzed using PlasmoDB, I-TASSER, and Alpha-Fold, which indicate a significant likelihood that certain genes encode membrane proteins and signal peptides. (B) Table of genes from panel A linked to the pt1 cluster, showing the PlasmoDB annotation and the presence (green) or absence (pink) of TM domains and/or signal peptides, with the top table panel showing the presence of both TM and signal peptides and with the bottom table showing the presence of either TM or signal peptide. (C) Table of genes from panel A linked to the pt2 cluster, showing the PlasmoDB annotation and the presence (green) or absence (pink) of a TM domain and/or signal peptides, with the top table panel showing the presence of both TM and signal peptides and the bottom table showing the presence of either TM or signal peptides. (D) Bar plot depicting the total percentage of disordered residues (*y* axis) in the predicted protein structures of the highly expressed, nonannotated genes (*x* axis). The color scale indicates the number of disordered segments present in each protein. The 3D structures were analyzed using the CSpritz server.

Due to the ability of membrane proteins to induce immune responses as well as their importance in nutrient/chemical uptake (35), we sought to define and characterize nonannotated membrane proteins, which make up approximately 33% of the *Plasmodium* genome (36). Such proteins could serve as targets in novel antibody-based therapies. First, from the pt clusters, we identified annotated and nonannotated genes that were significantly upregulated (adjusted *P* value of <0.05) (Fig. 6A). From pt1 and pt2, 41 and 111 nonannotated gene candidates made the described cutoff, respectively. The tables in Fig. 6B and C denote the presence or absence of a predicted transmembrane (TM) domain and/or a signal peptide. Interestingly, all the selected nonannotated genes in Fig. 6B and C were classified as intrinsically disordered proteins (IDPs), all of which had a minimum average log fold expression of 0.5 (Table S5). These proteins are characterized by the presence of large segments of disordered structure under normal physiological conditions. Proteins with distortion exceeding 15.5% of the entire protein secondary structure were classified as IDPs (Fig. 6D). IDPs have been described as highly immunogenic (37) and, in the case that they are demonstrated to be membrane associated and surface exposed, may be candidates for novel antibody- or peptide-based therapeutics. Given the abundance of IDPs in pt1 and pt2, we sought to investigate the stage specificity of expression of the genes predicted to encode IDPs by using the published Malaria Cell Atlas visual tool (https://www.sanger.ac.uk/tool/mca/mca/). Each of the 17 genes of interest (GOIs) was selected and analyzed in its expression across the UMAP representing the P. falciparum life cycle (Fig. S5). Taken together, we demonstrate the presence of IDP-encoding genes uniquely expressed under the midgut sexual stages. The general expression trend suggests that the majority of these genes are specifically expressed during the gametocyte or mosquito midgut developmental stage of the P. falciparum life cycle. Hence, these proteins make up a novel panel of GOIs that may be further analyzed for antibody-based transmission-blocking strategies.

## DISCUSSION

The transcriptional regulation of P. falciparum development in the mosquito midgut remains understudied, with the availability of only a single-cell atlas of P. falciparum mosquito stages that includes mature ookinetes (16). Using scRNA-seq, our data define the genetics underlying the differentiation of a female gamete to an ookinete. We characterized a progression of tightly regulated changes in gene expression driving P. falciparum development in the midgut lumen of A. gambiae, its natural mosquito vector. We also defined three distinct molecular signatures that orchestrate cell type transitions in the mosquito midgut. Genes related to DNA replication and metabolic processes were identified in early zygotes, and those related to reproduction, localization, and motility were identified in intermediate stages, while early to mid-ookinetes express genes related to entry into the host and to downregulation of metabolic processes. *Plasmodium* development in the mosquito involves a series of DNA replications and meiotic divisions. After fertilization, the zygote undergoes a round of DNA replication that increases ploidy to 4N, followed by a conventional two-step meiotic division (38). This is later followed by endomitotic division in the oocyst to produce the sporozoites. We observed the differential expression of genes involved in DNA replication and repair, as well as in meiosis, in three distinct expression programs: early, mid-, and late midgut development. The early program includes genes like *NEK2*, *NEK4*, and *MISFIT* that have been shown to regulate DNA replication in preparation for meiosis (39). The mid-developmental program includes genes like *SMC1*, *CAPD3*, and *RAD23*, involved in chromosomal segregation and DNA repair during meiosis (40–42). The late program includes proteins expressed exclusively in ookinetes like *SMC5*, as well as *RAD5*, involved in DNA replication and repair before and during mitosis (40, 43). Of interest, *PlasMei2*, which in Plasmodium yoelii has been shown to be expressed exclusively in the liver stage, where it controls chromosome segregation (44), was detected in the late program, possibly indicating differences in expression from P. yoelii. Thus, together with other late program genes, PlasMei2 may have a role during the mitosis of the oocyst sporogony. Differential gene expression analysis determined a single cellular lineage for isolated cells, most of which aligned with the time points of isolation, although a number of individual cells showed temporal transcriptional dynamics aligning with earlier or later time points. Interestingly, Seurat cluster 3 was largely composed of cells from the 4-h collection point, and the alignment of a significant portion of these cells as the root of the Slingshot pseudotime analyses leads us to speculate that cluster 3 contains cells linked to one or both of the following scenarios. The cluster may represent a subpopulation of cells that show the morphology of developing parasites that have arrested in development due to stress induced by detrimental factors from the blood bolus (i.e., human immune factors and mosquito microbiome) and therefore show an alternate transcriptional profile compared with proliferating parasites collected at the same time point. Additionally, a portion of nonfertilized female gametes may, after 4 h of development in the mosquito midgut, still present similar morphology as that of zygotes while in tandem showing a significantly altered transcriptome profile compared to the developing zygotes collected at the same time point. It will be interesting to experimentally characterize this in future studies, and it would be compelling to compare if similar transcriptional trends are found in parasites cultivated *in vitro*, compared with our *in vivo* analyses of parasite development inside the mosquito midgut.

In P. falciparum, most transcription factors belong to the Apetala2 (AP2) family, which consists of 27 genes. Some of these transcription factors are named according to the life cycle stage where they are expressed and exert their function, for example, *ApiAp2-g* and its role in gametocyte differentiation (45, 46). The progression through the life cycle in the mosquito midgut is dependent on the temporal regulation of ApiAP2 transcription factors, as indicated by the differential expression in pseudotime clusters. With the exception of *AP2-O4*, all *AP2-O* genes showed expression in our data set, and the high resolution provided by single-cell transcriptomics allowed the definition of the timing of *AP2-O* gene expression through the studied time course. Moreover, additional ApiAP2 genes appear to be upregulated in a significant proportion of cells (>50%). Two ApiAP2 genes were found in C2 (*PF3D7_1239200* and *PF3D7_1342900*) and four genes in C4 (*PF3D7_0420300*, *PF3D7_0934400*, *PF3D7_0802100*, and *PF3D7_1139300*), none of which had previously been linked to a developmental stage. In agreement with our data, *PF3D7_0934400* was shown to be highly expressed among ookinetes in the single-cell data by Real and colleagues (16). Also, *PF3D7_0622900*:*AP2Tel*, which is significantly upregulated in C4 in our data set, showed low to intermediate expression among ookinetes in the data set of Real and colleagues (16) and high expression among a portion of the oocyst-sporozoites. This could be indicative of its involvement in regulating DNA replication genes in oocysts. *AP2Tel* has been described to be expressed throughout the asexually replicating blood stage in P. falciparum, where it was shown to bind directly to telomeric repeats of all 14 chromosomes (47). In addition, *AP2Tel* (*AP2-SP3*) has been shown to have a role in sporozoite release from oocysts in P. berghei (10, 28).

The timing of ApiAP2-O expression coincides with the downregulation of three genes, *P25*, *H2B*, and *CPR*. These genes are known to be (i) a zygote surface protein (48), (ii) a component of the nucleosome known for packaging chromatin (49), and, interestingly, (iii) an antimalarial resistance-associated gene in sporozoites (50), respectively. The timing of expression of these genes raises the intriguing question of whether AP2-O may be involved in negative regulation of their expression. The involvement of AP2 transcription factors in the negative regulation of genes occurs in Toxoplasma gondii (51) but has only been speculated on for *Plasmodium* spp. In later time points, AP2-O upregulates an array of genes, among them *SOAP* and *WARP*, both important for ookinete stages and in invasion (26). Taken together, our results highlight the coordinated expression of ApiAP2 transcription factors during the developmental trajectory of P. falciparum in the mosquito midgut and beyond.

Since parasite numbers decline precipitously during mosquito midgut development, we also sought to elucidate the genetic program involved in this critical bottleneck of the P. falciparum life cycle. The parasite is afflicted by oxidative stress produced by free heme resulting from hemoglobin digestion, reactive oxygen species (ROS), toxic molecules produced by the midgut microbiome, human-derived immune and inflammatory molecules, and immune cells ingested with the blood meal (3). Subsequently, once the mature ookinete traverses the midgut epithelium, the mosquito complement immune response is activated, leading to increased susceptibility to nitric oxide toxicity (52). All of these factors contribute to the reduction of parasite numbers seen during the major life cycle bottleneck in the mosquito midgut (3, 8). Hence, we investigated the expression of genes involved in the response against ROS and reactive nitrogen species (RNS), cellular stress, and cell death. We found a temporal induction of known stress-related genes, indicating that different stressors from the mosquito midgut environment are associated with specific stress responses from the parasite. For example, the redox genes *nPrx*, *HSP90*, and *TCTP* were highly expressed in the intermediate stages (C1 and C0), likely to facilitate survival within the harsh midgut conditions. It has previously been reported that similar genes, such as *1-Cys peroxiredoxin* and *peroxiredoxin-1*, were upregulated in mosquito-derived P. berghei ookinetes compared with cultured ookinetes, due to the presence of ROS in the mosquito midgut and their role in protecting the parasite from oxidative stress (53).

Previous studies have used I-TASSER (54) to elucidate the structure of proteins of interest, including *in silico* experimentation for finding novel antimalarials (55). We used I-TASSER to predict protein structural annotation of a set of highly expressed, nonannotated genes linked to the mid- and late developmental points. Using three-dimensional (3D) model predictions, we found 17 nonannotated and highly expressed genes with a predicted TM domain and/or a signal peptide, indicating their possible membrane localization. We anticipate that these nonannotated genes can be further validated as potential targets for drug- or antibody-based interventions. There are currently multiple computational tools available to predict protein 3D structures as well as molecular docking sites, e.g., AlphaFold (protein structure) and DoGSiteScorer (56). Interestingly, the 17 nonannotated proteins were further indicated to be intrinsically disordered proteins (IDPs), proteins largely disordered in their structure and reported to be highly immunogenic (37). IDPs contain a number of traits that may elicit immunogenic and antigenic responses that have yet to be fully explored, though an interesting relationship between epitope processing, immunodominance, and protein disorder has been examined with cleavage in disordered sites leading to preferential presentation of adjacent epitopes (57).

The P. falciparum midgut stages represent a tenuous point in the parasite’s life cycle given the large decline in population size and genetic diversity. Such small numbers render the emergence of parasites resistant to therapeutics far less likely than in human stages. These properties make the *Plasmodium* mosquito stages a highly strategic point for malaria intervention. Drugs which target this stage will be highly specific against the parasite and can be administered to cure mosquitoes and indirectly prevent human infection (58). Further, human antibodies are potent, remain viable within the mosquito midgut, and are becoming affordable for use in low-income settings, indicating that transmission-blocking vaccines targeting the transmission stages of the parasite are possible and merit further exploration (9, 59, 60). Overall, our data provide an important resource for exploring the hitherto largely undescribed transcriptome of the mosquito midgut stages of Plasmodium falciparum and offer untapped potential for the exploration of transmission-blocking therapeutics.

## MATERIALS AND METHODS

### *In vitro* culture of P. falciparum parasites and gametocyte induction.

Gametocytes from the P. falciparum gametocyte-producing NF54 cell line were induced and cultured as previously described in reference 61. In brief, parasites were cultivated on type O-positive erythrocytes at 4% hematocrit in standard culture medium containing RPMI supplemented with 10% heat-inactivated human serum (Karolinska Hospital blood bank, Stockholm, Sweden) using standard culturing techniques. Cultures were gassed with 96% N_2_, 1% O_2_, and 3%CO_2_ and maintained on a shaking incubator at 37°C to prevent multiple infections in erythrocytes (RBCs). Gametocytes were then induced at 5 to 6% parasitemia and maintained with daily medium changes for 14 to 17 days in standard malaria parasite culture medium containing 25 mL of RPMI supplemented with 10% heat-inactivated human serum.

### Mosquito infections.

Two gametocyte cultures were harvested on day 17 and pooled. Gametocyte cultures were then diluted to 1% gametocytemia at 50% hematocrit and delivered directly into water-jacket glass-membrane feeders connected to a 37°C circulating water bath as standard procedure for each feeding assay. Female Anopheles gambiae mosquitoes (5 to 7 days old), maintained at 25 to 27°C and 80% humidity, were allowed to blood-feed for about 30 min, after which any unfed mosquitoes were isolated and discarded. Cages of 50 female mosquitoes were used for midgut collection divided over three consecutive time points. Thus, approximately 12 to 15 mosquito midguts from mosquitoes that had successfully taken a blood meal were dissected at time points 2, 4, 8, 12, and 20 h postinfection. Mosquito midguts were collected in 1.5-mL Eppendorf tubes with 500 μL of RPMI and homogenized using a microtube homogenizer (F65000-0000; SP-Bel-Art) at short intervals for 30 s. In addition, one batch of mature gametocytes was treated with aphidicolin (Sigma catalog no. A0781-1MG) *in vitro* and cultured for 2 h at room temperature. Aphidicolin prevents DNA replication in male gametes, rendering them immature, and thus blocks female fertilization.

### Staining and isolation of single parasites.

After midgut homogenization, 2 μL of anti-Pfs25 antibody (1 μg/mL) was added to the sample and incubated at room temperature (RT) for 15 min. One microliter of Alexa Fluor 488 goat anti-mouse IgG(H+L) at 2 μg/mL (Life Technologies; catalog no. A11001) and 0.5 μL of 10-mg/mL Hoechst 33342 were added to the sample and incubated for 15 min at RT in the dark. Cells were washed two times with sterile Dulbecco’s phosphate-buffered saline (DPBS; ThermoFisher Scientific; catalog no. A1285801), and parasite pellets were subsequently resuspended in 500 μL of DPBS. One hundred microliters of stained parasites was placed on bovine serum albumin (BSA)-coated petri dishes to prevent the adhesion of the parasite to the glass bottom of the petri dish. Target cells were visualized using a Leica DMi8 microscope (Leica, Germany) and collected using capillary-based micromanipulation with glass capillaries having an inner diameter of 8 μm (Eppendorf, Hamburg, Germany). Capillaries were precoated with a sterile 2% BSA solution to prevent the gametes from sticking to the glass capillary surface. Individual parasites were randomly captured based on the presence of green (Alexa Fluor 488) and blue (Hoechst 33342) fluorescence and the presence of an apparently intact cell membrane. Collected cells were replaced in 0.5 μL of 1× DPBS and transferred into 200-μL thin-walled PCR tubes (Corning, NY) containing 3.5 μL of lysis buffer (0.6% Triton X-100), 2 U/μL recombinant RNase inhibitor, 1 μL oligo(dT) (10 μM), and 1 μL deoxynucleoside triphosphate (dNTP) mix (10 mM). All samples were immediately stored at −80°C after isolation.

### cDNA synthesis and library preparation.

cDNA libraries of single parasites were generated using a modified version of the Smart-seq2 protocol (62). In short, cDNA synthesis was performed using P. falciparum optimized primers (17), and PCR amplification was carried out over 24 cycles. cDNA products were subsequently purified using CA beads (Sigma; catalog no. 81260) for size selection using 8.8% polyethylene glycol 6000 (PEG 6000) to exclude primer-dimers and nonspecific amplicons with sizes less than 150 bp. Combinatorial indexing via tagmentation was carried out in 96-well plates using 200 pg (measured in a Qubit fluorometer) of amplified cDNA, for a final volume of 10 μL/well. cDNA fragmentation using Tn*5* transposase was carried out for 20 min on ice using the Illumina Nextra XT DNA sample preparation kit. Ligation and amplification of adaptors were carried out over 15 cycles in a final volume of 25 μL/well. Primer indices were used in the reaction from Illumina (Nextera index primers i7 and i5, catalog no. FC-131-1001). Tagmented and barcoded amplicons were then purified using CA beads for size selection. Quality control and fragment size distribution of the cDNA libraries were performed on a Bioanalyzer with the Agilent high-sensitivity DNA chip (catalog no. 5067-4626). Concentrations of each sample of cDNA libraries were measured on a PicoGreen 96-well plate NucleoScan fluorometer using a high-sensitivity double-stranded DNA (dsDNA) (HS assay kit; catalog no. Q32851). To perform library dilutions, the average fragment sizes of all cDNA libraries were measured for a final concentration of 2 nM in each sample. Finally, cDNA libraries were pooled and sequenced using Illumina NextSeq with 75-bp paired-end reads.

### Computational analysis. (i) scRNA-seq raw data mapping and feature counts.

Raw scRNA-seq reads were processed and trimmed for quality and adaptor content using FastQC (version 0.11.5) (S. Andrews, 2012) and Trimmomatic (version 0.36) (M. Bolger, 2014), respectively. All quality-processed reads were mapped to human (GCF_000001405.38_GRCh38.p12_genomic.fna), mosquito vector (GCF_000005575.2_AgamP3_genomic.fna), and P. falciparum parasite (GCF_000002765.4_ASM276v2_genomic.fna) using FastQ_Screen, and the reads mapping to parasite but not human and vector were retained. All orphan unpaired reads were discarded, and remaining paired reads were mapped to teh P. falciparum genome downloaded from PlasmoDB (PlasmoDB-39_Pfalciparum3D7_Genome.fasta) using STAR (version 2.5.3a) (A. Dobin, 2013). Read quantification was performed using HTSeq-count (S. Anders, 2015) (parameters: -t exon -i gene_id -r pos -m intersection-nonempty), and custom bash script was used to generate the gene count matrix.

### Quality control and normalization.

For estimation of good-quality cells to perform downstream analysis, we used the Seurat package (v. 4.0.3) on the raw features count matrix for an overview of the distribution of the number of reads and genes detected per cell within each time point. We set a cutoff to filter out sequenced samples (single cells) with fewer than 600 genes detected and/or fewer than 32,000 reads, resulting in 125 cells that were included for the downstream analysis. We then employed a global-scaling “LogNormalize” to the feature expression measurements for each single cell from the total expression multiplied by a scale factor (10,000) with the normalized values stored in the Seurat object.

### Identification of highly variable features.

In order to delineate cell-to-cell variation across the data set and highlight the biological signal of highly expressed genes, we used the feature selection method “vst” implemented in the Seurat package (FindVariableFeatures) to find variable features across the single cells’ transcriptomes. The top 2,000 variable genes were selected for the downstream analysis.

### Dimensionality reduction, cell clustering, and projection.

To dissect cellular heterogeneity during the development of P. falciparum zygotes, we first scaled the expression so that the variance across cells had an equal weight of 1 in the downstream analysis. We then performed linear dimensionality reduction (principal-component analysis [PCA]) using the top highly variable genes (HVGs) on the normalized-scaled transcriptome data to characterize the variation across the data set. To estimate the number of PCs indicating a true signal of transcriptional variation, we visualized both the cells and features using the DimHeatmap function on the first 20 PCs and then performed the JackStraw test for the *P* value estimation. Next, we used the first 10 PCs (most PCs showing the signal variation) to delineate the transcriptome into cell type communities by running k-nearest neighbors (kNN) graph-based clustering and applying modularity optimization with the Louvain algorithm to relatively group the cells with a resolution of 0.8. Different cell types’ transcriptomes were then visualized using UMAP nonlinear dimensionality reduction. Detailed steps of the analysis are explained in the links under “Data availability.”

### Differential gene expression analysis.

To define markers distinguishing each cluster generated by the Louvain algorithm, we set a minimum percentage of cells expressing certain features to be detected at 25%. Finding differentially expressed genes across the cluster was set with a Logfc.threshold of 0.25 using the Wilcoxon rank sum test (false-discovery rate [FDR] of <0.05) with a minimum of 25% cells for cluster-specific markers comparing all remaining cells reporting positively and negatively regulated markers to be used in gene set enrichment analysis (GSEA) for particular biological pathway analyses. The differentially expressed gene data set is presented in Table S3 in the supplemental material.

### Cell lineage and pseudotime analysis.

Louvain clusters were ordered along the developmental trajectory using Slingshot (v1.8.0) (63). NFFGs were excluded, and the feature expression matrix was normalized and scaled to estimate the total mRNA abundance with the aid of the Seurat object tool along with metadata previously generated from Louvain clustering. To restructure cell lineage and develop a pseudotime inference for the purpose of uncovering the global mRNA structure of the single-cell data set, we used the minimum spanning tree (MST) algorithm and fit our cells simultaneously against a “principal curve” to establish mRNA distribution in an unsupervised manner. Applying the MST recovers a single lineage of developmental trajectory composed of three global cell communities (pseudotime regulatory modules). Based on differentially expressed features recovered using the FindAllmarkers function, specific cluster markers were identified and were assigned as rooting cells (C3) or the lineage starting point (initial cells) of the developmental trajectory while the pseudotime values were estimated simultaneously for each single cell ordered along the fitting curve. To visualize the pseudotime values specifically for the Seurat clustering and the isolated time points, we overlaid the single cells’ metadata on the Slingshot lineage to have a better understanding of the relationship between the clusters and the isolated time points (Fig. 2B). To find genes that change their expression over the course of development, we used the Tradeseq package 1.4.0 (64) to calculate the relationship between gene expression and pseudotime. In brief, we used a general additive model (GAM) to model the relationship between genes and conduct an association test to estimate the *P* value of genes significantly expressed over time (Fig. 2C and Table S4). We summarized snapshot transcripts of the global structure lineage, and notable GO terms from PlasmoDB.org were used to distinguish pseudotime gene coexpression modules (pt0 to pt2). To assess genes transcriptionally regulated during the course of development with significant variation in their expression, we employed the global clustering structure forming the Slingshot single lineage.

### Motif identification for ApiAP2 regulators.

We explored known ApiAP2 target sequence motifs using a PlasmoDB search on transcriptionally regulated genes generated from pseudotime analysis (differentially expressed genes along the pseudotime), which returned only the genes with a specific known binding mix-base motif within a 1-kb upstream region of the starting codon for each ApiAP2 transcription factor target gene. Next, we visualized the target gene coexpression dynamics in relation to the specific transcription factor showing the regulation of target genes over the course of pseudotime data from reference 26.

### Biological pathways and GSEA.

To track tests for top functional class enrichment among the global clusters building the pseudotime lineage, we used conservative markers generated for each cluster on the PlasmoDB GO analysis tool to conclude the enriched ontology terms as previously mentioned. The gene set enrichment analysis (GSEA) was performed on differentially expressed genes over the pseudotime as input in cluster profiler v 3.18.1 and ggupset package v 0.3.1 with a *P* value cutoff of 0.05, minGSSsize of 3, maxGSSize of 800, and scoreType of “pos” to estimate for biological process ontology changes over the pseudotime lineage and developmental progress (Fig. S4A and Table S2). The top 20 biological processes were visualized using the Clusterprofiler package (v 3.18.1) dot plot function.

### Protein prediction analyses.

The amino acid sequences of significantly upregulated, nonannotated genes of P. falciparum 3D7 were retrieved from the PlasmoDB website and processed for their primary structures and physicochemical properties. Briefly, Expasy’s ProtParam tool (65) was utilized to calculate the physicochemical characteristics and the secondary structural properties including α-helix, 310 helix, Pi helix, beta bridge, extended strand, bend region, beta turns, random coil, ambiguous states, and other states using the Multivariate Linear Regression Combination tool, together with the Self-Optimized Prediction Method with Alignment tool (MLRC/SOPMA) (66). I-TASSER (54) was used for the structure-based functional annotations. Predicted proteins with high-confidence prediction annotations were then examined for the presence of signal peptide or TM domains. Alpha-Fold (protein structure database) was utilized to retrieve the .pdb files of the predicted protein structures (67). I-TASSER Modrefiner was used for structure refinement (68) based on atomic-level energy minimization.

### Identification of IDPs.

We computationally characterized the 3D homology structures of protein models showing a lack of well-characterized protein segments (intrinsically disordered proteins [IDPs]) using the CSpritz server for accurate detection of protein disorder (69) in combination with comparative dynamic simulations with Python-based docking libraries (py3Dmol, openbabel, and nglview). Briefly, .pdb files were retrieved from Alpha-Fold and imported in the Jupyter notebook with perquisite libraries imported and compared with well-structurally annotated proteins. We then identified the characteristics of bulky hydrophobic amino acid sequences with high net charges promoting disorder in the form of extended loop regions coupled with folding and binding compared to the core structure, resulting in instability and irregularity of the secondary structure (70).

### Data integration of 24-h ookinetes (day 1) from the work of Real et al.

Expression matrices and supporting metadata files were downloaded from https://zenodo.org/record/4719664/. Single-cell data were subset according to the target stage (day 1 ookinete). Variable genes were intersected between the two data sets using Scanpy (v1.5.0) (F. A. Wolf, 2018). We then used the concatenate and ingest function to integrate data annotations and labels and corrected for batch effect using BBKNN integrated in the Scanpy workflow.

### Data availability.

In-house bash, R code scripts, and data that were implemented in this study are available on GitHub (https://github.com/ANKARKLEVLAB/Single-cell-P.falciparum-midgut). Expression matrices and metadata are available via https://zenodo.org/record/4683823#.Y7wZaOzMJQZ, and the data are also searchable via https://mubasher-mohammed.shinyapps.io/shinyapp/. The raw data is deposited in the Gene Expression Omnibus (GEO) database, accession number: GSE222586.
