# Supplementary material for: Single-Cell Transcriptomics To Define Plasmodium falciparum Stage Transition in the Mosquito Midgut
Source: Microbiol Spectr. 2023 Feb 27;11(2):e03671-22. doi: 10.1128/spectrum.03671-22 (PMC10100735; doi:10.1128/spectrum.03671-22)
Supplement: Supplemental file 7 — Fig. S1-S5. Download spectrum.03671-22-s0001.pdf, PDF file, 3.5 MB [file spectrum.03671-22-s0001.pdf]

Supplementary Figure 1

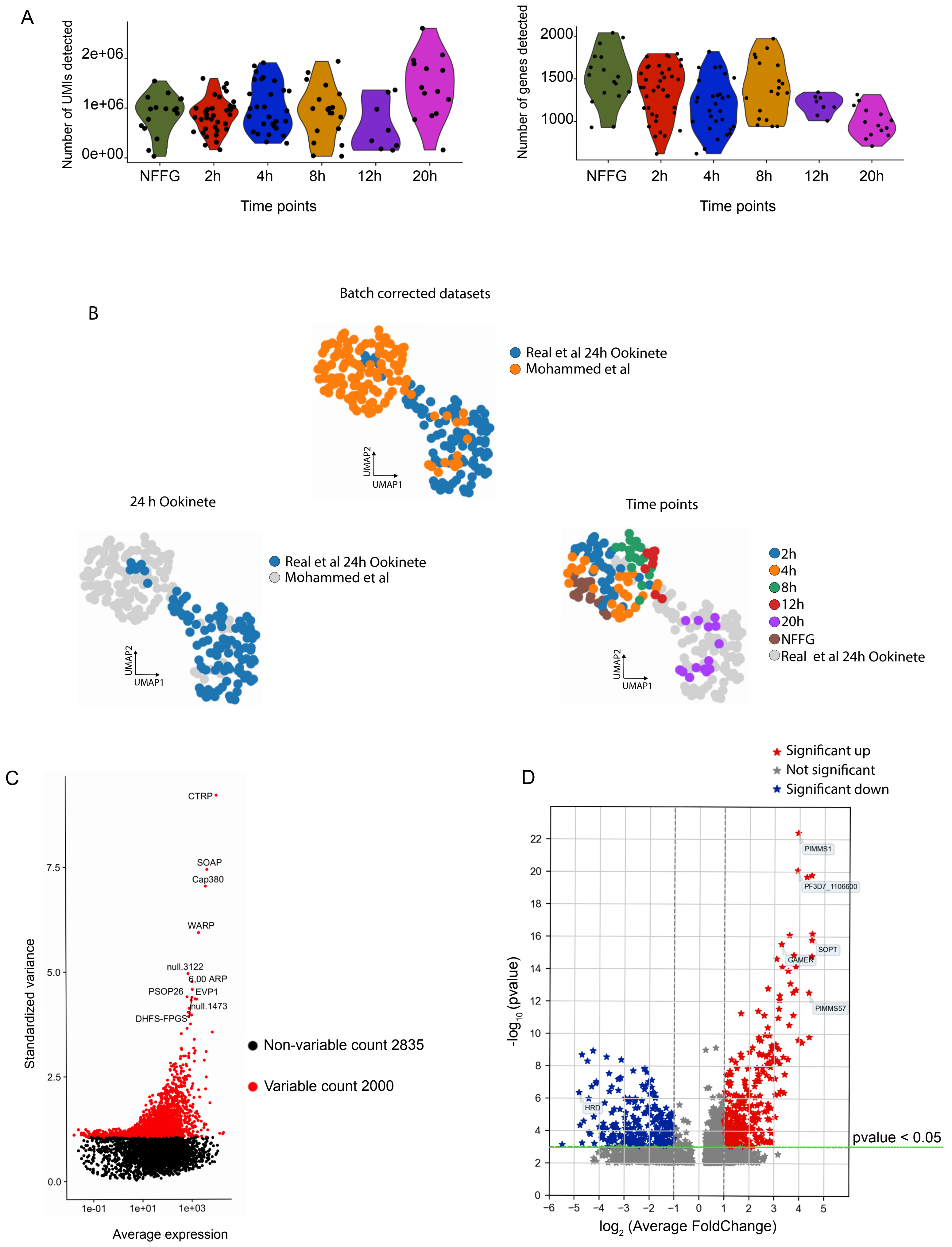

Supplementary Figure 2

A

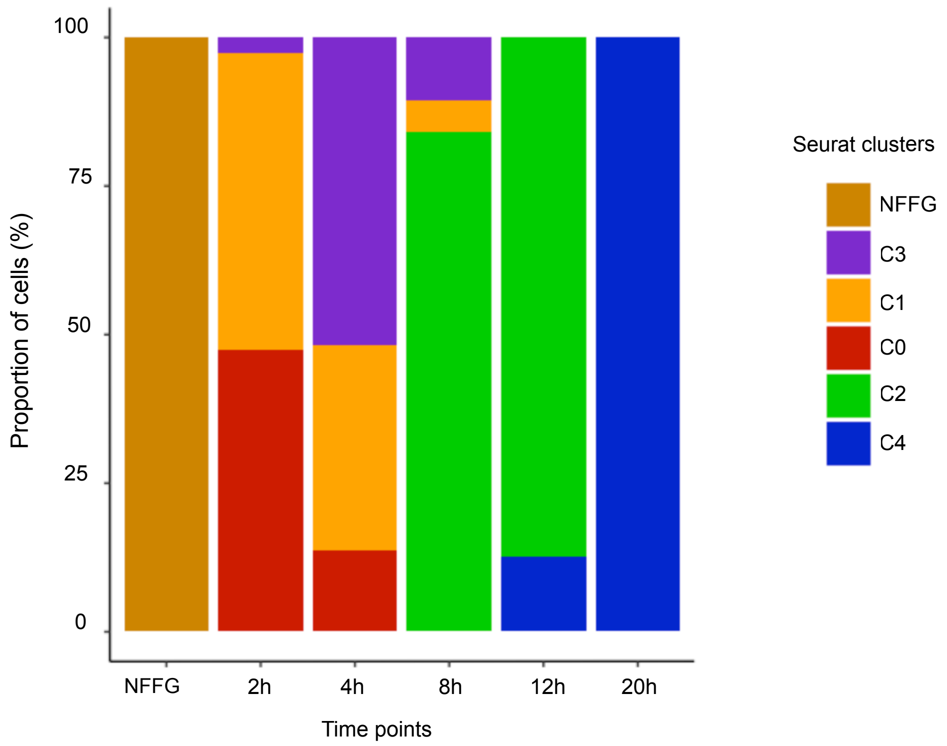

B

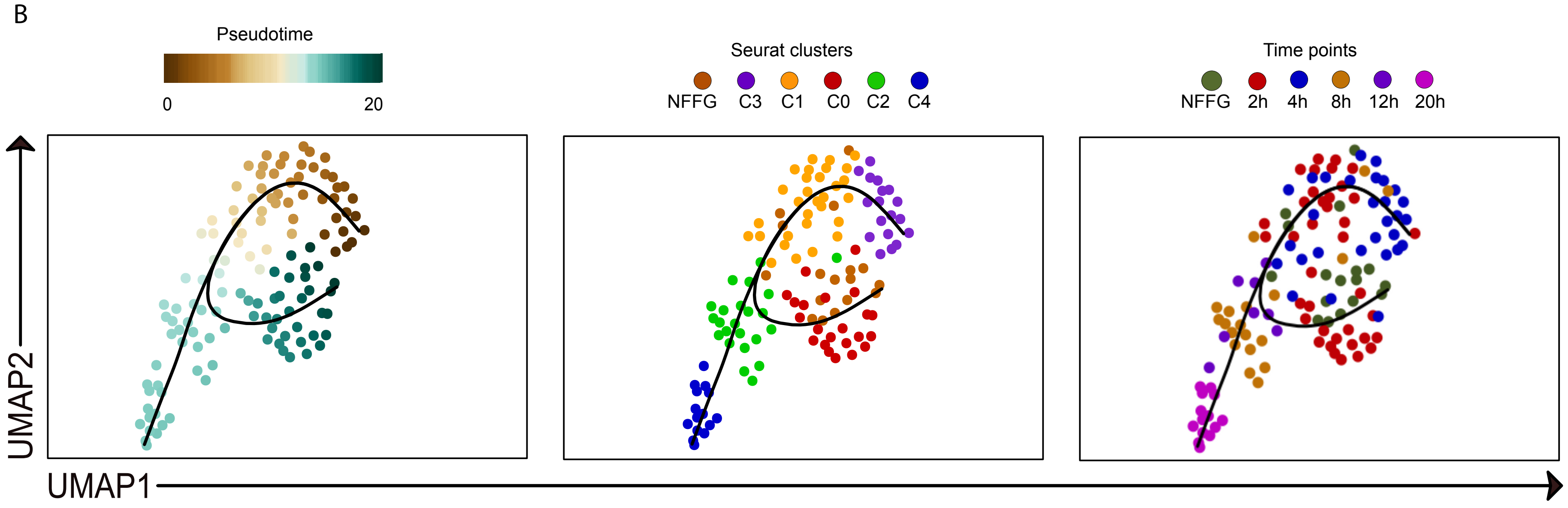

Supplementary Figure 3

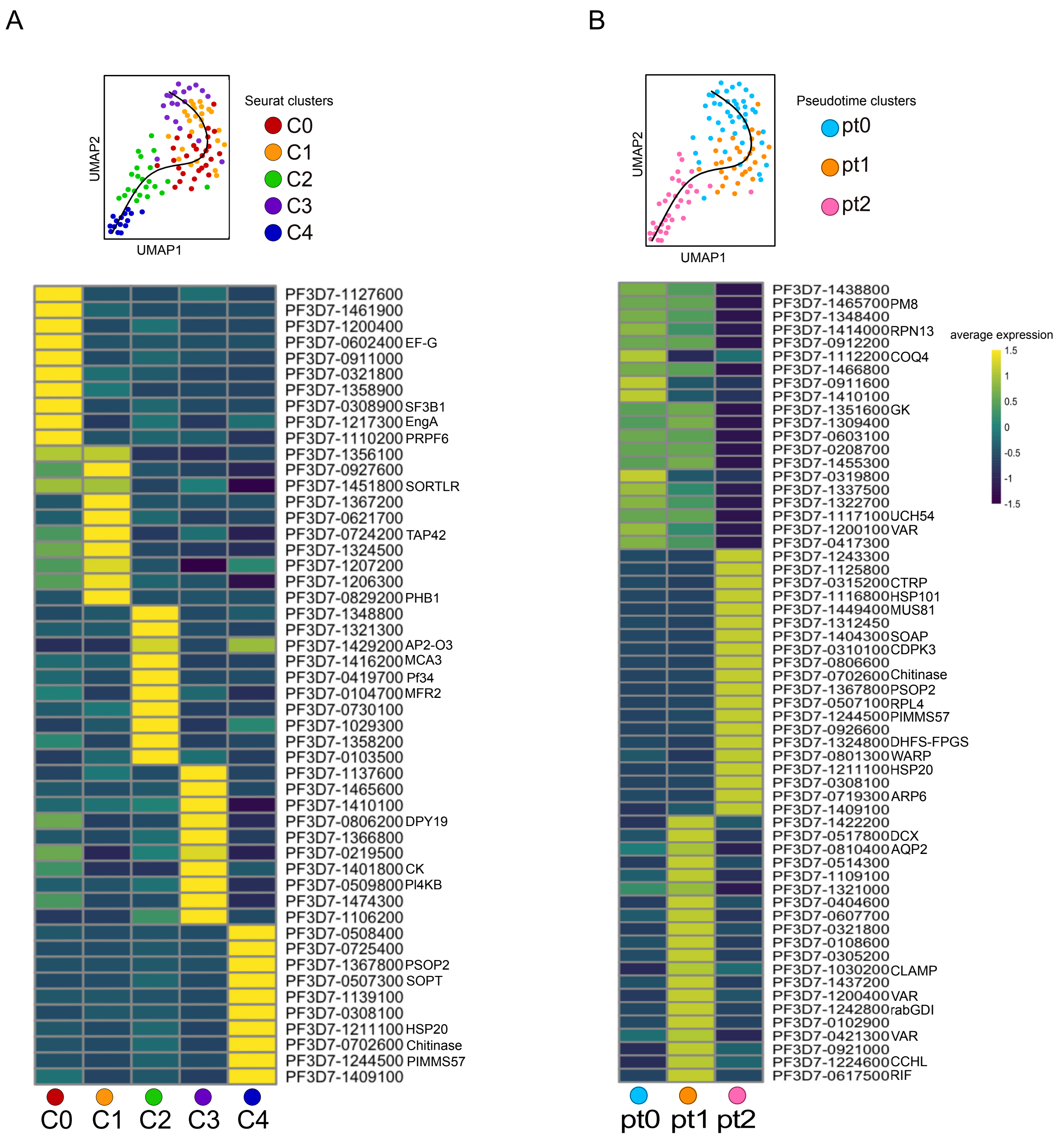

Supplementary Figure 4

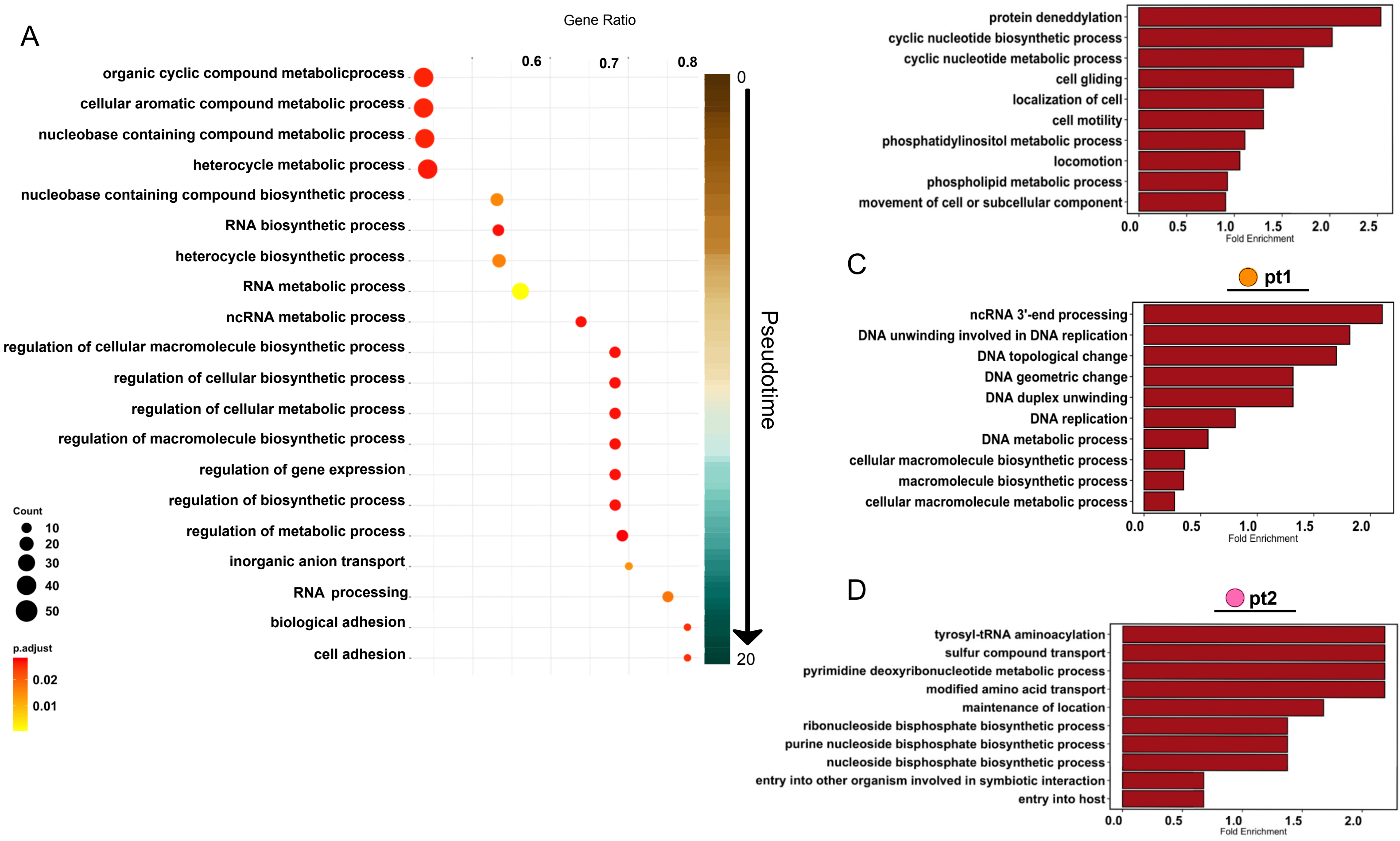

Supplementary Figure 5

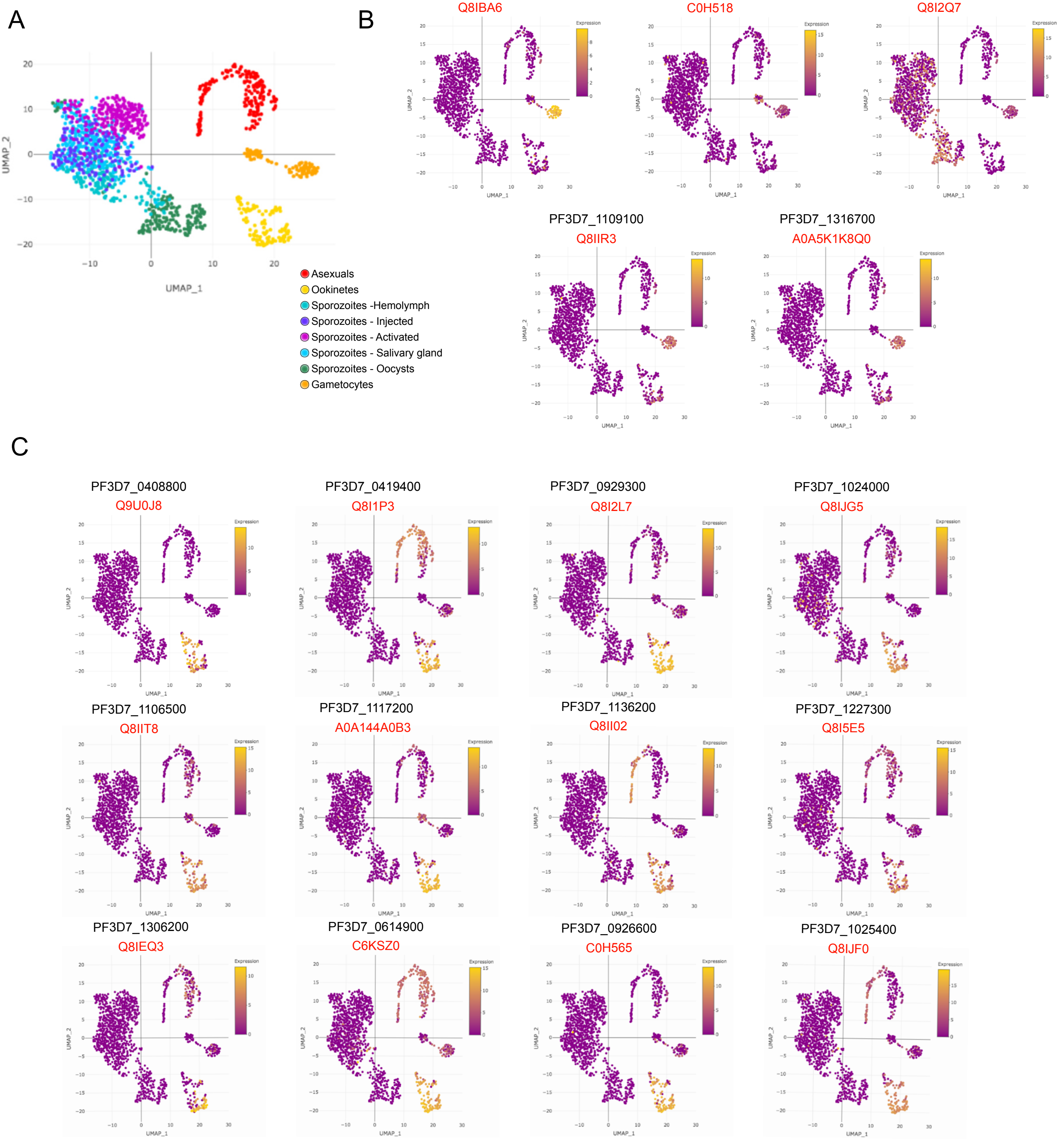

Supplementary Figure Legends

Supplementary Fig 1. Quality assessment of Smart-seq2 data and data integration with Real *et al*. 2021

A. Violin plots showing the number of unique molecular identifier (UMI)/transcripts detected per cell (Left panel) and the number of genes detected per cell (Right panel) across the six collection timepoints. **B**. Data integration of Real *et al.* 2021, day 1 (24h) single-cell transcriptomes with time points determined in this work non-fertilized female gametes (NFFG) to 20 h single-cell transcriptomes. Upper panel: shows integrated datasets. Bottom panel left: integrated datasets highlighting (blue) 24h ookinetes from Real *et al*. Bottom panel right: integrated datasets highlighting the isolated time points determined in this work. After batch correction, more single cells from this work represent early development of *P. falciparum* in mosquito midgut stages. **C**. Volcano plot depicting the most highly variable genes, with the top 10 genes labelled. A total of 2,835 features were identified as non-variable while 2,000 features were variable and selected for downstream analysis. **D**. Volcano plot showing differentially expressed genes, based on Seurat cluster comparison, color-coded as red for significantly up-regulated, blue for significantly down-regulated, or Grey for not significant. The x-axis represents the fold change while the y-axis green dashed line represents the *P*-value ($fdr < 0.05$). A total of 1,576 differentially expressed genes were identified

Supplementary Fig 2. Comparison of the Slingshot trajectory analysis in the presence or absence of the non-fertilized female gametes (NFFG)

A. Bar plot showing the ratio of Seurat clusters identified within each collection time point. **B.** The UMAPs indicate a modified trend of the Slingshot pseudotime trajectory

when NFFGs are included in the analysis as compared with the trajectories shown in Figure 2A, where the inclusion of the single-cell transcriptomes from NFFGs appears to distort the global lineage reconstruction. Left panel: The alternative pseudotime trajectory where the color coding represents the cells predicted development along the pseudotime axis. Middle panel: The pseudotime developmental trajectory overlaid with the assigned Seurat clusters and where NFFGs are separately color coded in brown. Right panel: The pseudotime developmental trajectory overlaid with the cell isolation timepoints and including NFFG.

Supplementary Fig 3. Comparison of top differentially expressed genes between the Seurat and Slingshot clusters along the pseudotime trajectory

A. The Slingshot pseudotime developmental trajectory overlaid with the Seurat clusters (top) with a hierarchical clustering heatmap showing the top 10 differentially expressed genes for each individual cluster (bottom). **B**. Slingshot pseudotime developmental trajectory overlaid with the Slingshot pseudotime clusters (pt0, pt1, pt2) (top) with a hierarchical clustering heatmap of the top 20 differentially expressed genes for each individual cluster (bottom). The Wilcoxon rank test was used in the differential gene expression analysis (for both A and B) of genes significantly expressed ($fdr < 0.05$) in at least 25% of cells and a logFC threshold of 0.25.

Supplementary Fig 4. Gene ontology enrichment by Slingshot clustering

A. Dot plot of a gene set enrichment analysis from the top differentially expressed genes over the pseudotime. Genes of interest were grouped according to the top 20 GO terms identified in the three pseudotime clusters and statistically analyzed by gene ratio with more significantly (red) and less significantly (yellow) expressed genes, color

coded and organized by gene count (dot size). The X-axis indicates gene ratio, and the Y-axis indicates GO terms. **B**-**D**. Bar plots of top GO terms showing the over-represented GO terms for three (pt0, pt1, pt2) cell communities determined using Slingshot pseudotime curve reconstruction. Top 10 GO terms were selected per cluster.

Supplementary Fig 5. Comparison of highly expressed non-annotated genes identified in this study with the malaria cell atlas (MCA)

A. UMAP showing single-cell transcriptome colored according to *P. falciparum* life cycle stages. **B**. Expression profiles of highly expressed non-annotated genes identified in the pt1 cluster on UMAP of *P. falciparum* single-cell transcriptome. **C**. Expression profiles of highly expressed non-annotated genes identified in the pt2 cluster on UMAP of *P. falciparum* single-cell transcriptome.
